# Canavan Disease as a Model for Gene Therapy-Mediated Myelin Repair

**DOI:** 10.3389/fncel.2021.661928

**Published:** 2021-04-23

**Authors:** Anoushka Lotun, Dominic J. Gessler, Guangping Gao

**Affiliations:** ^1^Horae Gene Therapy Center, University of Massachusetts Medical School, Worcester, MA, United States; ^2^Department of Microbiology and Physiological Systems, University of Massachusetts Medical School, Worcester, MA, United States; ^3^Department of Neurosurgery, University of Minnesota, Minneapolis, MN, United States; ^4^Li Weibo Institute for Rare Diseases Research, University of Massachusetts Medical School, Worcester, MA, United States

**Keywords:** Canavan disease, NAA, myelination, oligodendrocyte, astrocyte, white matter, leukodystrophy, gene therapy

## Abstract

In recent years, the scientific and therapeutic fields for rare, genetic central nervous system (CNS) diseases such as leukodystrophies, or white matter disorders, have expanded significantly in part due to technological advancements in cellular and clinical screenings as well as remedial therapies using novel techniques such as gene therapy. However, treatments aimed at normalizing the pathological changes associated with leukodystrophies have especially been complicated due to the innate and variable effects of glial abnormalities, which can cause large-scale functional deficits in developmental myelination and thus lead to downstream neuronal impairment. Emerging research in the past two decades have depicted glial cells, particularly oligodendrocytes and astrocytes, as key, regulatory modulators in constructing and maintaining myelin function and neuronal viability. Given the significance of myelin formation in the developing brain, myelin repair in a time-dependent fashion is critical in restoring homeostatic functionality to the CNS of patients diagnosed with white matter disorders. Using Canavan Disease (CD) as a leukodystrophy model, here we review the hypothetical roles of N-acetylaspartate (NAA), one of the brain's most abundant amino acid derivatives, in Canavan disease's CNS myelinating pathology, as well as discuss the possible functions astrocytes serve in both CD and other leukodystrophies' time-sensitive disease correction. Through this analysis, we also highlight the potential remyelinating benefits of gene therapy for other leukodystrophies in which alternative CNS cell targeting for white matter disorders may be an applicable path for reparative treatment.

## Introduction

Leukodystrophies, also known as myelin or white matter (WM) disorders, result from functional mutations in glia or downstream metabolic divergence in neuronal and/or non-neuronal populations (Seitelberger, 1984; Powers and Rubio, 1995; Kevelam et al., 2016). These genetically rare myelin diseases are often characterized in early childhood with several clinical presenting forms, namely hypotonia, ataxia, and seizures (Traeger and Rapin, 1998; Barkovich, 2005; van der Knaap and Bugiani, 2017). Some symptoms have also been observed of advancing into more severe stages such as hydrocephaly and megalencephaly (Matalon et al., 1988; Bugiani et al., 2010; Desai and Grimm, 2013; Sánchez et al., 2020). Consequently, many diagnosed patients can progressively develop significant debilitation and cognitive impairment, thus negatively affecting their quality of life and contributing to emotional and financial burdens on their families. Abating and conceivably eradicating leukodystrophies remains a central focus of the scientific and healthcare communities. Recent studies have aimed to target glial cells, which appear to be the primary affected cell types, as an avenue for prospective leukodystrophy therapy (Muramatsu et al., 2007; Piguet et al., 2012; Kagiava et al., 2014; Francis et al., 2016; Georgiou et al., 2017; Gessler et al., 2017; Jonquieres et al., 2018). However, several key challenges are posed in this course of treatment. The first lies in tailoring therapy specifically toward the type of myelin abnormality in the leukodystrophy; i.e., either hypomyelination, a deficit of myelin; demyelination, a degradation of myelin; or dysmyelination, defective myelin (Naidu, 1999; van der Knaap and Bugiani, 2017), which may have relevance in the strategies used for therapeutic treatment. The second concerns the current limitations and challenges of efficiently and selectively targeting certain glial populations, particularly oligodendrocytes, in the brain to enable normalization of the myelin disorder. A third takes into consideration the potential contribution of peripheral organs in treatment strategies for leukodystrophies as little is currently known about how other organs play a role in its disease etiology. Irrespective of these challenges however, treatment is thought to be geared toward correcting for myelin deficits observed in affected individuals. Here we review the demyelinating progression of Canavan disease (CD) as an example of prospective avenues for leukodystrophy therapies, as well as the current approaches, setbacks, and successes of gene therapy as a time-dependent corrective measure for this lethal demyelinating leukodystrophy.

## Etiology of Canavan Disease

Characterized as a rare leukodystrophy, Canavan disease makes for an applicable and apt disease to examine the inner workings between demyelination and gene therapy application in a basic and translational setting. CD is caused by a loss of function of the aspartoacylase (ASPA) gene primarily in ASPA-expressing mature oligodendrocytes of the Central Nervous System (CNS) (Kirmani et al., 2002; Moffett et al., 2007). The resulting dysfunctional ASPA enzyme inhibits oligodendrocytes from hydrolyzing neuronally derived N-acetylaspartate (NAA), one of the brain's most abundant amino acid derivatives, into acetate and L-aspartate. This leads to the disease's characteristic accumulation of NAA in the CNS (Matalon et al., 1988; Toft et al., 1993), detectable by elevated NAA peaks in proton magnetic resonance spectroscopy (H-MRS) (Toft et al., 1993). The increased NAA concentrations observed in CD contrasts that of other leukodystrophies, such as Vanishing White Matter (VWM) disease (Bugiani et al., 2010) and Alexander disease (AD) (Moffett et al., 2007; Vázquez et al., 2008), which have been reported to instead present with a reduction of NAA (Moffett et al., 2007; Ariyannur et al., 2013). Nonetheless, in relation to its potential myelinating relevance, NAA levels in the CNS indirectly serves as a biomarker for neuronal health as well as a potential building block for CNS metabolism (Moffett et al., 2007; Francis et al., 2016; Jonquieres et al., 2018).

## Hypothetical CNS Functions of NAA

To date there is limited understanding of the connection between non-functional NAA hydrolysis and myelin deterioration in Canavan disease. Perhaps the closest, indirect relationship has been seen phenotypically wherein NAA levels increase upon degradation of the WM (Leone et al., 2012; Francis et al., 2016). Although no conclusive consensus on the role of NAA currently exists, five leading hypotheses have been proposed in recent years to better understand its function in the CNS as well as its potential myelinating relevance in CD (Baslow, 2002; Moffett et al., 2007, 2013; Pederzolli et al., 2007, 2009; Kołodziejczyk et al., 2009; Jonquieres et al., 2018).

### NAA-Derived Acetate Used for Lipid Synthesis

The first hypothesis suggests that in a healthy brain, NAA-derived acetate is used as a building block for fatty acid synthesis. *In vivo* research performed by Benuck and D'Amado et al. demonstrated supporting evidence for this hypothesis in which they traced radiolabeled NAA in rat brains, revealing that the liberated acetate from catabolized NAA was used in generating long chain fatty acids (Benuck and D'Adamo, 1968). Supplemental *in vitro* research performed on immortalized brown adipogenic cells (iBACs) over-expressing the NAA synthesizing enzyme, N-acetyltransferase 8-like (NAT8L), depicted a 4-fold increase of incorporated labeled-glucose into lipids such as triacylglycerols and free fatty acids (Pessentheiner et al., 2012; Huber et al., 2018), thus further reinforcing this hypothesis. In the context of Canavan disease, wherein NAA cannot be broken down into its subcomponents, NAA accumulation leads to an acetate deficiency and thus a decrease in lipid production (Madhavarao et al., 2005). This hypothesis in part suggests that the generated lipids from NAA catabolism could be used for myelin formation, which therefore would account for the demyelination in CD (Namboodiri et al., 2006).

### NAA-Derived Acetate Used in Energy Metabolism

Consequently, the acetate provided from NAA breakdown has also been hypothesized to be involved in energy production. In this hypothesis, acetate would be synthesized into acetyl-CoA *via* acetyl-CoA synthase for its subsequential use in the citric acid cycle (D'Adamo et al., 1968; Namboodiri et al., 2006). In CD however, acetate production from NAA is compromised, which therefore would lead to an insufficient acetate pool for acetyl-CoA generation (Ariyannur et al., 2010). It could be hypothesized that acetate supplementation could possibly correct this feature, however, acetate replacement studies, by way of oral glyceryl-triacetate (GTA) administration in the CD tremor rat model and the traumatic brain injury rat model, showed limited therapeutic benefits; although improvements in motor performance and vacuolization were observed (Mathew et al., 2005; Arun et al., 2010; Moffett et al., 2013), complete normalization was not achieved, suggesting that other pathological factors may be at play. Accordingly, in the acetate deficient environment of CD, the affected CNS may require an alternate source of energy, which could potentially be provided from lipid degradation. Supporting i*n vitro* studies using iBACs over-expressing NAT8L demonstrated a 40% elevation in the basal oxygen consumption rate compared to control brown adipocytes (Pessentheiner et al., 2012), suggesting that NAT8L overexpression, and therefore NAA accumulation, increases cellular respiration and consequently energy production potentially through lipolysis (Pessentheiner et al., 2012; Huber et al., 2018). An extension of this hypothesis would also indicate that the expenditure of fatty acids for energy production results in a reduction of myelinating lipids.

### NAA Toxicity Affects Myelination

The third hypothesis proposes that NAA is toxic and therefore its elevated concentration in the CNS may lead to downstream progressive demyelination and vacuolization. Several groups have generated supporting data for this hypothesis in that increased oxidative stress on proteins, impaired non-enzymatic antioxidant defenses (Pederzolli et al., 2007, 2009), and absence-like seizure activity (Kitada et al., 2002) were reported following direct stereotaxic intracerebroventricular injections of NAA into rat brains. Patch-clamp studies further back these findings by demonstrating that NAA and its anabolic counterpart N-acetyl-aspartyl-glutamate (NAAG) depolarize gray matter granule cells *via* N-methyl-D-aspartate (NMDA) receptors in a calcium induced manner, but to a much lesser extent in white matter oligodendrocytes (Kołodziejczyk et al., 2009). Thus, NAA-mediated stimulation of neuronal NMDA receptors could serve to explain the seizure activity reflected in the previous study. Correspondingly, if elevated NAA concentrations are considered to be cytotoxic, one could hypothesize that NAA, or likewise NAAG, could result in NMDA receptor overstimulation in either neurons or oligodendrocytes and therefore cause cell death, thus accounting for demyelination in CD. However, the same group, Kolodziejczyk et al., has shown contrasting data on this front, depicting that upon incubation of cerebellar slices in the presence of NAAG, non-significant differences in cell death were observed in comparison to the control group.

In addition to *in vitro* experiments, *in vivo* studies have also depicted a toxic vs. non-toxic duality role of NAA. Rodent models in which both alleles for the NAA synthesizing enzyme, NAT8L, were knocked down revealed restorative pathology with a lack of CNS vacuolization (Maier et al., 2015) and a visible reduction of astrogliosis (Guo et al., 2015), contrasting that of CD rodent models expressing NAT8L. However, the survival and motor performance of these animals remained untherapeutic (Guo et al., 2015; Maier et al., 2015), suggesting that complete knockdown of NAA synthesis is not sustainable long-term. Likewise, other studies have shown that ASPA deficiency in rats causes seizure activity, reminiscent of the CD phenotype, which is subsequently reduced upon ASPA restoration *via* gene therapy (Klugmann et al., 2005), thus further supporting this hypothesis. On the contrary, heterozygous NAT8L expression in mice resulted in almost fully sustained survival comparable to wildtype (WT) animals, however, complete remedial reversal of CNS spongy degeneration was not observed (Maier et al., 2015). Other groups such as Jonquieres et al. have used animal lines overexpressing NAT8L to demonstrate that NAA elevation does not cause neuropathology, and thus does not intrinsically contribute to neurotoxicity, hypothesizing that it is rather the compartmentalization of NAA in oligodendrocytes, specifically the lack of oligodendroglial NAA hydrolysis and intolerance to NAA buildup in oligodendrocytes, which contributes to CD pathology (Jonquieres et al., 2018). Interestingly, concurrent research using cultured stomach cells extracted from rat tissue was found to exhibit nitric oxide toxicity through dose-dependent NAA incubation, in which upregulation of nuclear factor-kappa B (NF-kB) and mitogen activated protein kinases (MAPKs) were observed, leading to activation of nitric oxide synthase (iNOS) and cell death mediation, respectively (Surendran, 2009, 2010). Although the duality of whether NAA is toxic or non-toxic is still unclear, if the finding of nitric oxide toxicity holds true for the CNS, it could be hypothesized that elevated NAA in ASPA deficient oligodendrocytes causes toxicity through a potentially similar pathway.

### NAA-Derived Acetate Used for Epigenetic Regulation

The fourth theory bridges the utilization of NAA-derived acetate for energy production to epigenetic regulation (Prokesch et al., 2016) and considers a potential role of peripheral organs in CD pathology. The hypothesis follows that acetyl-CoA generated from acetate precursors are used in transcriptional control *via* acetylation of histone groups (Takahashi et al., 2006; Moffett et al., 2013). Auxiliary research done by Prokesch et al. demonstrated that *in vitro* ASPA knock-down in brown adipocytes led to a significant reduction of histone lysine acetylation, particularly of H3K27ac and H3K9ac (Prokesch et al., 2016), raising the question if the periphery plays a role in CD gene regulation and/or if the CNS undergoes similar epigenetic changes which may lead to downstream pathological effects. In this manner, Canavan disease which presents with an inadequate amount of NAA-derived acetate, could result in altered epigenetic regulation given that acetyl-CoA levels would be reduced due to a lack of available acetate pools, thus consequently altering gene expression.

### NAA Acts as an Osmolyte in the CNS

Lastly, according to the molecular water pump (MWP) theory, NAA has also been hypothesized to function as a neuronal osmolyte involved in osmoregulation (Baslow, 2002). Given the CNS vacuolization observed in some severe forms of CD, the MWP hypothesis aims to provide an association between CNS edema and the role of NAA in maintaining water homeostasis (Baslow and Guilfoyle, 2013). Studies using microdialysis have previously shown NAA levels increase in the extracellular fluid (ECF) of rat brains in a stepwise manner following perfusion with increasingly hyposmotic media, thus suggesting that NAA is released as a result of osmotic changes which contribute to downstream extracellular swelling (Taylor et al., 1995). However, this would suggest that NAA is potentially transported out of neurons and subsequently into the extracellular matrix, which has not yet been determined by scientific research (Moffett et al., 2007).

## NAA's Functional Correlation in Astrocytes

In the main hypotheses presented for NAA's myelinating role in the CNS, we see a common thread of liberated acetate from NAA catabolism acting as a precursor for lipid synthesis and energy production. This is interesting because as evidenced by radiolabeled tracing of acetate, astrocytes have previously been identified as acetate transporters from the extracellular space into synaptosomes (Waniewski and Martin, 1998). In addition, they have been noted to be one of the major utilizers of acetate, oxidizing it for downstream use in the TCA cycle (Waniewski and Martin, 1998; Moffett et al., 2013). Therefore, following the hypothetical role of NAA-derived acetate in lipid production for myelin synthesis as well as the role of astrocytes in regulating acetate, it would be possible that astrocytes are fundamental for remyelination in the context of Canavan disease therapy.

Interestingly, the MWP theory also proposes a dynamic and possibly metabolic use of astrocytes, rather than oligodendrocytes, in normalizing NAA levels (Baslow and Guilfoyle, 2009). In this regard, astrocytes, which are known to form critical, regulatory gap junctions with oligodendrocytes (Benfenati and Ferroni, 2010; van der Knaap and Bugiani, 2017), could be the key to restoring myelination in CD. The premise follows that elevated NAA levels in the CNS are in part due to catabolism of NAAG *via* NAAG peptidase (Baslow and Guilfoyle, 2009) located on astrocytes. This subsequently increases osmotic pressure following water influx toward regions of amplified NAA concentration, which is hypothesized to result in the hallmarked WM edema observed in CD (Baslow, 2002; Baslow and Guilfoyle, 2009). In addition, water transport proteins such as aquaporin 4 (AQP4) have been found to be localized in astrocyte foot processes and play important regulatory roles in maintaining water homeostasis and osmolality in the CNS (Baslow and Guilfoyle, 2009; Papadopoulos and Verkman, 2013; Clarner et al., 2014). Electron microscopy (EM) studies of CD patients further reinforces these scientific findings by depicting large vacuole formation in cortical astrocytes (Adachi et al., 1972), effectively characterized as astrocytic swelling (Moffett et al., 2007; Baslow and Guilfoyle, 2009). Collectively, the NAAG-derived NAA and myelinic edema in CD alludes to the partaking of astrocytes in NAA metabolism and the resulting downstream pathology of vacuolization. This observation is markedly significant because like oligodendrocytes, astrocytes also contribute to CNS myelination (Nash et al., 2011).

## Pathobiology of Canavan Disease

The phenotypic changes attributed with hallmarked demyelination in CD, such as ataxia, muscle weakness, and motor impairment, are shared amongst other leukodystrophies (Moers et al., 1991; Traeger and Rapin, 1998; van der Knaap and Bugiani, 2017). Notably, these changes are representative of the downstream effects of non-functioning myelin and thus neuronal impairment. In CD, magnetic resonance imaging (MRI) and diffusion tensor imaging (DTI) testing has shown ASPA deficient brains to have white matter abnormalities, which can histologically present as cerebral and cerebellar spongiform vacuolization (Adachi et al., 1973; Toft et al., 1993; Matalon and Michals-Matalon, 2000; Barkovich, 2005; Traka et al., 2008; Sommer and Sass, 2012; Gessler et al., 2017). Because myelin constitutes an integral role in the CNS, its degeneration can cause lasting adverse effects such as a delay in or loss of neuronal function due to decreased axonal conductivity and/or cellular metabolism, both of which are critical for neuronal survival (Philips and Rothstein, 2017). This in turn is supported by brain biopsies of CD patients which exhibit significant neuronal loss in deep cortical regions (Adachi et al., 1973; Matalon and Michals-Matalon, 2000). The consequential, and observed, neuronal debt can clinically present in the form of seizures, cognitive impairment, and weakened psychomotor responses. Thus, to better understand the downstream pathology of neuronal deterioration, we must first discern the upstream effects of demyelination in Canavan disease.

## Glial Composition of White Matter

Collective pathological features between CD and other leukodystrophies requires a brief account into the nature of CNS myelination. An appreciation for the complexity of cell-type connectivity throughout the brain is central in further characterizing the framework of myelin and the supportive role of glial populations in its maintenance. As research has shown, WM is constructed from an array of glia, mainly comprised of but not limited to oligodendrocytes, oligodendrocyte precursors cells (OPCs), and astrocytes (Back et al., 2002; Young et al., 2013; van der Knaap and Bugiani, 2017). Wherein oligodendrocytes construct myelin, astrocytes are responsible for maintaining oligodendrocyte and neuronal homeostasis (Philips and Rothstein, 2017; Jorge and Bugiani, 2019). One particular example of this is the astrocyte-neuron lactate shuttle, in which, upon the presence of glutamate binding, astrocytes uptake circulating blood glucose and convert it to pyruvate (through glycolysis), and subsequently into lactate by lactate dehydrogenase (LDH). The generated lactate is then shuttled, *via* monocarboxylate transporters (MCT), into neurons and oligodendrocytes where it is converted back into pyruvate for energy production through the Kreb's cycle, thus maintaining the functional, energic needs of those respective cell populations (Pellerin and Magistretti, 1994; Kasischke et al., 2004; Philips and Rothstein, 2017). This close-knit homeostasis between the major CNS cell-types is indicative that WM integrity, and therefore neuronal health, is contingent upon a coordinated equilibrium of glial cell types (Casper and McCarthy, 2005). This suggests that leukodystrophy treatments in the case of CD may not be entirely reliant on simply rescuing oligodendrocytes, but rather may involve a re-balancing of overall glial function to restore or correct for myelin loss.

## Genetic and Time-Dependent Significance of Myelination in Canavan Disease

Myelination in the developing brain is a concerted effort of differentiating oligodendrocyte precursor cells (OPCs) into mature, myelinating oligodendrocytes (Barres et al., 1992; Trapp et al., 1997). However, the rate of OPCs differentiation varies during the human lifespan, with turnover into myelination peaking within the first 2 years of life and slowing down soon after (Barkovich, 2005). In the human forebrain, oligodendrocyte transcription factor 1 (Olig1) and platelet-derived growth factor receptor alpha (PDGFRα) positive cells can appear as early as 6 weeks into gestational development and begin differentiating into myelin basic protein (MBP)-expressing mature oligodendrocytes at 8 weeks of gestational age (Jakovcevski et al., 2009). Past neonatal age however, OPC's rate of differentiation decreases (Back et al., 2002), potentially as a result of increased brain size and the need for more focused regulation of glial proliferation (van der Knaap and Bugiani, 2017). This distinction is noteworthy because it highlights the complexity of myelin formation during development. In addition, characterization of CD neuropathology suggests that the demyelinating pattern that contributes to the disease progression is time dependent (Smith, 1973).

Originally characterized by Adachi et al. (1973), the clinical onset of Canavan disease was classified into one of three major forms; infantile, congenital, and juvenile. Although genetically, based on current scientific knowledge, all forms of Canavan disease are congenital (Mendes et al., 2017), the infantile and juvenile classifications serve to demonstrate the variability in clinical presentations of the disease. The infantile form, which is most common type and often results in the most severe progression (Adachi et al., 1973; Matalon et al., 1988; Traeger and Rapin, 1998), displays no phenotypical abnormalities at birth. However, within the first months of life, patients begin to present with hypotonia and delayed cognitive responses (Matalon and Michals-Matalon, 1999). These symptoms can progressively develop into more severe, potentially lethal, forms of psychomotor arrest and hydrocephaly (Matalon et al., 1988; Matalon and Michals-Matalon, 2000). The juvenile type presents later in the patient's lifespan, at roughly around 5 years of age (Adachi et al., 1973). Patients with this form typically survive into late adolescence and exhibit a milder phenotype of the disease (Traeger and Rapin, 1998).

Notably, the varying degrees of clinical severity in Canavan disease patients, either severe or mild, are, respectively, contingent upon the genetic makeup and developmental stage of the disease. This is further backed by a recent study of a group of 14 patients with novel ASPA missense mutations which demonstrated that ASPA mutations exhibiting <1% of enzymatic activity tended to correlate with a more severe disease development and an early onset of phenotypic presentations including increased mental delay, increased motor delay, and lack of speech (Mendes et al., 2017). On the contrary, patients with ASPA mutations that resulted in roughly 10% of wildtype ASPA's enzymatic activity were associated with a juvenile, milder presenting form of the disease (Mendes et al., 2017). However, this distinctive genotype and phenotype correlation did not pertain to all patients in the study. One patient, classified as a mild case, had normal language development and motor control, yet harbored an ASPA mutation that rendered <1% of enzymatic activity. A second mild patient had an ASPA mutation with ~12% of enzymatic activity, which was considered in the study to be a relatively high functionality, yet presented with severe clinical phenotypes such as mental delays and language impairment within the first 3 months of life (Mendes et al., 2017). This observed independent phenomenon was also demonstrated in a separate clinical study in which two children were classified with juvenile, late onset forms of CD but similarly had low enzymatic ASPA activity, 0 and 5%, respectively (Leone et al., 1999). An analogous case was also seen in two additional CD patients who had the genetic A305E ASPA mutation associated with a severe form of CD, but presented with the juvenile course of the disease (Shaag et al., 1995). Interestingly, it has also been shown that the enzymatic activity of ASPA in four non-affected, heterozygous carrier parents of Canavan disease patients was 40% of the WT ASPA (Barash et al., 1991), further compounding efforts to properly correlate the genotypic and phenotypic relationship in Canavan disease. Nonetheless, the several cases of associated clinical presentation to enzymatic activity do suggest that, to a certain extent, an existing ASPA mutation in an individual Canavan patient may affect whether the disease will present in either an infantile or juvenile manner (Adachi et al., 1973; Barkovich, 2005; Mendes et al., 2017). In this regard, a component of genetics and time are essential in fostering a deeper understanding of the myelination pathogenesis and timeline for treatment intervention in Canavan disease. A distinction of the genetics, pathological onset, and severity of Canavan disease is critical because, like other leukodystrophies, therapeutic efforts, and success will in part rely on correcting for the developmental CNS progression of myelination in a time-sensitive manner (Lattanzi et al., 2010), which is further challenging due to the varying developmental forms of CD (Best et al., 2018; Knaap et al., 2019). Thus, conducted CD research would need to account for disease advancement in the context of successive CNS myelination.

## Rodent Models for Canavan Disease

Several CD rodent models have been developed in recent years, allowing for further understanding of the demyelinating disease progression. By way of positional cloning techniques, the tremor rat model (*tm/tm*) was constructed by genomic deletion in the region containing the ASPA gene, resulting in lack of ASPA expression and presence of phenotypical Canavan disease traits such as CNS spongiform degeneration (Kitada et al., 2002). This rat model has also been used to study myelin lipid abnormalities in CD (Wang et al., 2008). However, one caveat of the *tm/tm* rat model is that other genes, most notably the calcium-calmodulin-dependent protein kinase IV gene, which is involved in calcium signaling, are located in the same deletion region as the ASPA gene, thus potentially creating complications in studying single gene contribution to disease pathology. On the other hand, the *nur7* and CD KO mouse models, both of which only effect the ASPA gene, enable for research into more distinct gene-specific etiology. The *nur7* mouse model, first screened by Kile et al. and further classified by Traka et al., encodes a nonsense mutation, Q193X, in the ASPA gene resulting in a non-functional, truncated protein (Kile et al., 2003; Traka et al., 2008). The resulting ASPA^nur7^ mutant mice parallel the mild form of Canavan disease in that they phenotypically exhibit elevated NAA levels, spongy degeneration, and vacuolization in the CNS, but do not develop early lethality from disease progression. Contrastingly, the ASPA knockout mouse model (CD KO), first constructed in a C57BL/6 background (Matalon et al., 2000) and, due to genetic instability, was later crossed into a SV129/Ev background (Ahmed and Gao, 2013; Ahmed et al., 2016; Gessler et al., 2017), presents with the severe form of Canavan disease, manifesting acute disease progression, such as reduced motor function, spasticity, and lethality as early as 4 weeks of age. Interestingly, CD KO mice in the C57BL/6 background did not consistently display the severe phenotype found in the SV129/Ey background. Both the CD KO and ASPA^nur7^ mutant mice mirror the phenotypic nature of Canavan disease and thus use of these mouse models allows for more applicable readouts of CD gene therapy effectiveness. The ASPA^nur7^ mutant mice, which exhibit milder phenotypes and longer lifespans, enable investigations into potential successes of long-term gene therapy, which is specifically beneficial to study adult CNS myelination. The CD KO mice, on the other hand, exhibit severe phenotypes consequently followed by a shortened lifespan and therefore permit research into immediate readout of gene therapy efficacy, particularly at neonatal, juvenile, and young adult timepoints. The time-dependent pathology onset in these CD mouse models can accordingly provide further insight into CD neuropathology which may aid in disease regression and prevent potential fatality associated with acute forms of the disease.

## Glial Cell Targeting For Leukodystrophies Using Gene Therapy

Combating the high lethality rates of leukodystrophies such as Canavan disease has been a challenging feat for the scientific community. Due to a lack of genetic screening, challenges for current prognoses are augmented (Lattanzi et al., 2010; Best et al., 2018; Knaap et al., 2019), thus further complicating effective diagnoses at early postnatal ages. Thus far, treatment for CD has mainly centered around symptomatic remedies rather than curative therapies, with the exception of several clinical trials, as later discussed. Nonetheless, recent implementations of CNS-directed gene therapy using Lentiviruses (LVs) and recombinant Adeno-Associated Viruses (rAAVs) have opened novel therapeutic paths, particularly for Canavan disease.

Lentiviral use for *in vivo* gene therapy is notable for its high transgene capacity (Kagiava et al., 2014), long-term sustainability of transgene expression, and ability to integrate into post-mitotic cells (Ashrafi et al., 2019). Previous research has illustrated its therapeutic application for leukodystrophies such as globoid cell leukodystrophy (GLD) (Lattanzi et al., 2014) and metachromatic leukodystrophy (MLD) (Piguet et al., 2012). Despite these successes however, LV has been reported in both adult rodents (Alisky et al., 2003; Lundberg et al., 2008) and in non-human primates (Kordower et al., 2000) for its limitations in non-neuronal capsid tropism. Intending to overcome these challenges, promoters such as myelin-basic protein (MBP) and 2,3-cyclic nucleotide 3-phosphodiesterase (CNP) have been used in mice for more directive oligodendrocyte expression, but transduction rates for this cell population remains as low as 20–30% past juvenile ages (Kagiava et al., 2014). Therefore, given its predominantly neuronal tropism and reduced glial transduction capabilities, LV application may not be as clinically relevant for leukodystrophies such as Canavan disease, which may require more robust glial expression at early developmental stages.

Adeno-associated virus has, in recent years, been the preferential gene delivery vector for CNS related diseases. Out of the now continuously growing library of serotypes present in the gene therapy field, several prominent AAVs have been thoroughly identified in displaying notable CNS transduction capabilities, contingent on the route of administration, in the mouse brain, mainly AAV2, 7, 8, 9, and rh.10 (Davidson et al., 2000; Burger et al., 2004; Taymans et al., 2007; Cearley et al., 2008; Yang et al., 2014). Of these serotypes, AAV2 exhibits the lowest transduction efficiency (Cearley et al., 2008; Yang et al., 2014) with a primarily neuron-tropic profile (Kaplitt et al., 1994; Xu et al., 2001). Contrastingly, AAV8 and AAV9 are established for having more widespread CNS transduction (Muramatsu et al., 2007; Cearley et al., 2008) particularly at neonatal stages, but nonetheless have a similar preferential neuronal selectivity to that of AAV2 (Lawlor et al., 2009).

Established clinical trials for CD patients first began through intraventricular delivery of liposome-encapsulated plasmid DNA (LPD) of pAAV-ASPA in a group of first two CD patients and then later in 14 CD patients (Leone et al., 1999, 2000). In this phase I trial, it was found that dose administration of 80 μg/mL in a total LPD-complex volume of 5 mL provided partial reversal of NAA levels, although hyperintensive T2-weighted imaging depicted that these patients still exhibited cases of CNS spongiform degeneration post-treatment (Leone et al., 2000). Advancements in gene therapy at the time subsequently allowed for utilization of AAV2, known for its neuronal tropism, as the new CNS transfer vector for the neuron-specific enolase (NSE) driven ASPA transgene (Janson et al., 2002; Leone et al., 2012). The 13 CD patients chosen for the study had six burr holes drilled intracranially to allow for placement of infusion cannulas for CNS AAV-ASPA delivery of 1 × 10^12^ GC/mL in a total volume of 150 μL per injection site (Leone et al., 2012). Longitudinal post-treatment studies over the course of 48 months revealed a broad decrease in NAA concentrations and an overall mass increase of subcortical brain regions in these patients (Leone et al., 2012). Immune response studies from the phase I CD trial showed 30% of subjects developed neutralizing antibodies (NAB) to AAV post-gene therapy, but nonetheless maintained a relatively basal and consistent, unchanging level of pro-inflammatory cytokines, suggesting the relative safety following rAAV administration (McPhee et al., 2006). Clinical monitoring of other CD patients has also shown a regional and partial decrease in previously elevated NAA concentrations as well as indications of slowdown of brain mass reduction upon gene therapy treatment (Hoshino and Kubota, 2014). Undoubtedly, the success of rAAV administration on these CD patients portrays the potential gene therapy has in the context of leukodystrophies. However, as discussed, clinical trials using the AAV2 vector presents limitations in CNS targeting of non-neuronal cell types. In the scope of leukodystrophy gene therapies, greater glial transducing AAVs for clinical trials would enable effective targeting of other glial cells. Although hypothetical, further research into widespread CNS tropic AAVs such as AAV9 may present with more advanced clinical progress for leukodystrophies.

Irrespective of vector tropism, promoters such as MBP, myelin-associated glycoprotein (MAG), and glial fibrillary acidic protein (GFAP) have been utilized to enhance oligodendrocyte and astrocyte targeting (Muramatsu et al., 2007; Lawlor et al., 2009; Jonquieres et al., 2013, 2016; Georgiou et al., 2017; Gessler et al., 2017). The difference in vector choice is evidenced by research done comparing EGFP expression levels in adult mice treated with MBP driven EGFP using a lentiviral vector, which yielded 20% oligodendrocyte transduction (Kagiava et al., 2014) vs. the same construct packaged in the AAV which yielded 25 and 95% transduction in neonates and juvenile mice, respectively (Jonquieres et al., 2013; Kagiava et al., 2014). Therefore, use of promoters such as MBP, MAG, and GFAP, in conjunction with AAV capsids expressing global CNS tropism, such as AAV8 and 9, provides a greater ability in targeting selective glial populations.

In the context of gene therapy, existing literature has thus far presented effective oligodendrocyte transduction in the brains of adult and juvenile murine and rodent models (Jonquieres et al., 2016; Georgiou et al., 2017). In CD, however, this oligodendrocyte approach presents with a major setback in that current gene therapies are limited in their ability to selectively target oligodendrocyte populations during neonatal development. This is showcased by MBP driven EGFP transgene expression in neonatal, juvenile, and adult mice in which the MBP promoter leads to high leaky astrocyte expression in neonates which is consequently reversed upon treatment in juvenile mice (Jonquieres et al., 2013). Given the progressive myelinating pattern in the developing brain as well as the increasing postnatal rate of myelin dysfunction in affected individuals, it may be imperative to consider therapies applicable at early ages, especially for leukodystrophies which can have an infantile or juvenile onset.

## Gene Therapy Success in Mouse Models for Canavan Disease

Interestingly, recent research suggests that ubiquitous targeting of CNS cell types using intravenous systemic delivery of rAAV9 packaged with the chicken beta-actin (CB) driven hASPA transgene in mice treated at postnatal day 1 (P1) is adequate for rescuing CD KO mice and furthermore leads to sustained motor and histological recovery into adulthood (Gessler et al., 2017). Likewise, use of the gene therapy vector AAV/Olig001 carrying the ASPA transgene has demonstrated effective reversal of Canavan disease pathology to wildtype levels, such as reduced thalamic and cerebellar vacuolization and increased myelination, as well as normalization of NAA levels upon predominately oligodendrocyte-specific targeting (Francis et al., 2016, 2021). Additional research as also shown promising disease rescue and remyelinating success in the cerebellum, hippocampus, and thalamic CNS regions of ASPA KO mice treated with an AAV cy5 serotype packaged with the oligodendrocyte-specific MBP promoter driven ASPA transgene (Jonquieres et al., 2018). Notably, both the AAV/Olig001 and AAV cy5 gene therapy successes for Canavan disease models were achieved *via* direct stereotaxic injections into the CNS (Francis et al., 2016, 2021; Jonquieres et al., 2018). In the context of route of administrations, systemic rAAV delivery to achieve widespread CNS transduction is challenged by the blood brain barrier, which affects dose requirements and thus the side-effect profile of therapeutic efficiency. On the other hand, direct intracranial injection allows for a more local delivery of rAAV which may change the intraparenchymal transduction spread. However, from a clinical and translation standpoint, direct brain injections can be invasive medical procedures and therefore further research is needed to address the therapeutic effects of alternate, perhaps more systemic, routes of administration for oligodendrocyte targeting in the context of Canavan disease reversal in clinical trials.

More surprisingly, current data has also shown that selectively expressing ASPA in astrocytes, *via* rAAV9 GFAP-driven hASPA, can phenotypically rescue CD KO mice, also treated at P1 by systemic injections, to wildtype levels (Gessler et al., 2017). In these treated animals, histological data and behavioral tests, respectively, confirm normalized CNS pathology, such as lack of spongiform degeneration, intact myelination, and complete motor restoration. In addition, use of the GFAP promoter, which has high astrocytic selectivity (Lawlor et al., 2009; Gessler et al., 2017), possesses the added advantage of robust transduction in the rodent brain. This is notable because astrocytes are the most abundant CNS cell type (Haydon and Carmignoto, 2006; Casper et al., 2007) and given their role in myelin maintenance and CNS metabolism (Baslow and Guilfoyle, 2009), they are a probable cell type for using gene therapy to effectively target the CNS on a global scale. For leukodystrophies such as Canavan disease this is important because the effects of demyelination can be far reaching. Thus, for late stage myelin deterioration which may require more robust disease turnaround, targeting astrocytes could be beneficial given their largescale CNS distribution.

## Prospective Astrocytic Roles in Pathobiology of Canavan Disease

Astrocytes, which are known for their homeostatic role in the CNS, are critical participants in maintaining and regulating metabolic functions in the brain (Pellerin and Magistretti, 1994; Kasischke et al., 2004). As previously discussed, research pertinent to the role of NAA has depicted the involvement of astrocytes in acetate regulation and NAAG catabolism, thus linking a mechanistic feature of astrocytes to the CD pathomechanism. In addition to these components, astrocytes have also been reported to affect other CNS factors such as neuronal-glial signaling by way of glutamate-mediated NMDA receptors (Kasischke et al., 2004), and changes in myelinating capabilities of oligodendrocytes by modulating the extracellular matrix (ECM) (Pellerin and Magistretti, 1994; Nutma et al., 2020). Both of these aspects may hold relevance for CD therapies and help to rationalize the restorative and sustained success met with astrocyte-directed gene therapy in CD mouse models.

In fact, literature has shown that astrocytes encompass a duality function, both destructive and protective, toward oligodendrocyte pathology (Tilborg et al., 2016). Research suggests that the local environment of oligodendrocytes, in part controlled by mitogen release by astrocytes, affects the proliferation rate of myelinating oligodendrocytes (Barres and Raff, 1993; Young et al., 2013). For example, *in vitro* studies have shown that astrocytes operate as paracrine regulators by secreting factors such as platelet-derived growth factor (PDGF) in response to TNFα and IL-1β pro-inflammatory cytokines, which act to suppress oligodendrocyte differentiation (Gard et al., 1995; Silberstein et al., 1996; Zhang and An, 2007). On the contrary, *in vivo* studies in mouse models of other demyelinating diseases such as Multiple Sclerosis have demonstrated that reactive astrocytes secrete stromal cell-derived factor 1 chemokine 12 (CXCL12) to promote oligodendrocyte differentiation (Patel et al., 2012). In addition to cytokine signaling, glutamate signaling, which is required for proper oligodendrocyte myelination (Fannon et al., 2015), is regulated through expression of glutamate transporters (Goursaud et al., 2009) and NMDA receptors (Lalo et al., 2006) on astrocytes to prevent excitotoxicity. Furthermore, recent research using the cuprizone-induced demyelinating model has found that deletion of voltage gated Cav1.2 calcium channels in GFAP-positive astrocytes reduces astrogliosis and promotes remyelination, observable by a significant increase of Olig2-positive proliferating cells and a significant decrease in Iba1 and GFAP expressing cells (Zamora et al., 2020). Considering the downstream metabolism and catabolism of NAAG to NAA, as well as the importance of glutamate and calcium channels in astrocytes, it is possible that ASPA gene therapy in astrocytes somehow normalizes glutamate or calcium and/or cytokine pools in the CNS, thus correcting CD pathology; i.e., either directly by modulating glutamate or calcium levels to prevent hyperexcitability of neurons in an environment otherwise devoid of sufficient myelination, or indirectly by altering mitogen release necessary for oligodendrocyte growth.

Although there is not yet a clear explanation as to why and how astrocyte-targeted gene therapy can rescue CD pathogenesis and reverse demyelination in CD KO mice, it nonetheless raises into question how dependent Canavan disease treatment is on targeted oligodendrocyte populations. From a demyelination standpoint, the success of selective astrocytic therapy has noteworthy implications because it hints at the potential cellular crosstalk between either solely glial populations or inter neuronal-glial roles that allow for rescue of CD KO mice. This is supported through *in vivo* work that has shown a preferential ability of oligodendrocytes to remyelinate neuronal axons which are surrounded by astrocytes (Blakemore and Crang, 1989; Franklin et al., 1991; Talbott et al., 2005). *In vitro* research done by Nash et al. has also depicted that reactive astrocytes can induce myelination (Nash et al., 2011), thus reinforcing the notion that astrocytic restoration of ASPA could be beneficial in CD due to its ability to aid in remyelination, possibly through NAA-acetate homeostasis. Similarly, the hypothesized metabolic sink theory, in which NAA is conceivably redirected to astrocytes (Gessler et al., 2017) likewise supports the potential role astrocytes play in NAA metabolism, and thus in myelination. Interestingly, astrocytes have also been explored as therapeutic targets for other neurological diseases.

## Therapeutic Benefits of Astrocytic Targeting in Other Neurodegenerative Diseases

As previously discussed, astrocytes have both a restorative and degenerative role in the CNS (Tilborg et al., 2016). Fine tuning of their metabolic and functional regulation, such as is the case of CD, has shown to be similarly favorable for neurodegenerative disorders such as Amyotrophic Lateral Sclerosis (ALS) and Rett Syndrome. Treatments for ALS, which is a progressively degenerative disease affecting motor neurons and neighboring astrocytes in the brain and spinal cord, are linked to mutations in the superoxide dismutase (SOD1) protein which has recently shown therapeutic promise from astrocyte targeting (Valori et al., 2019). In SOD1 transgenic mice, mutant SOD1 astrocytes which are prone to mediated cell death by way of glutamate-activated metabotropic type-5 receptor (mGluR5), depict a deceleration of disease progression upon mGluR5 inactivation (Rossi et al., 2008). This receptor blockage in astrocytes extends animal survival and decreases astrocytic degeneration, further prolonging ALS disease onset (Rossi et al., 2008). Similar ALS research has depicted that downregulation of intracellular calcium following mGluR5 activation in mutant SOD1 astrocytes can likewise extend survival and improve motor function of SOD1 mice (Martorana et al., 2012). Astrocytes' important neurological roles are also observed in Rett syndrome mouse models. Rett Syndrome is caused by mutations in the X-linked methyl CpG-binding protein 2 (Mecp2) gene, which primarily affects neurons and subsequently adjacent glia (Chahrour and Zoghbi, 2007). Research in the past decade has found that re-expression of Mecp2 in astrocytes of Mecp2 knockout mice restores motor function, prolongs the mice lifespan, and improves phenotypic presentations of the disease (Lioy et al., 2011). As in the cases of ALS and Rett Syndrome, we observe a common functionality of astrocyte reprogramming leading to amelioration of disease progression, similar to the success of astrocyte-selective gene therapy in CD. These findings suggest that treatment for other neurological diseases and leukodystrophies, which can result from a variety of affected cell types (van der Knaap and Bugiani, 2017), may be achieved through alternative cell-type targeting.

## Alternative Cell Targeting Strategies for Leukodystrophies

Advances in stem-cell therapies in recent years have also shown promising therapeutic results for Canavan disease. Using Canavan patient derived induced pluripotent stem cells (iPSCs) to differentiate into either neural progenitor cells (iNPCs) or oligodendrocyte progenitor cells (iOPCs), it has been elegantly demonstrated that upon stereotaxic injection of iNPCs or iOPCs into brains of *nur7* mice, reduced NAA, reversal of spongiform degeneration, prolonged motor function restoration, and significantly improved myelination are observed (Feng et al., 2020).

In addition to stem-cell therapies, gene therapy for leukodystrophies such as Metachromatic Leukodystrophy (MLD) and Krabbe's disease (KD) have also illustrated the therapeutic benefit of targeting unconventional cell types relative to the core of the pathology. MLD, which results from a deficiency of the lysosomal enzyme arylsulfatase A (ARSA), is a leukodystrophy which exhibits neurological damage with similar phenotypes to that of CD, such as ataxia and seizures, in addition to early infantile or juvenile death (Neufeld, 1991; Biffi et al., 2006). The ARSA enzyme is highly expressed in the white matter of the CNS and cerebellum as well as the glands of the endocrine system and is responsible for converting sulfatides, a sphingolipid, for its use in cell membranes. Notably, sulfatide has been shown to be involved in axon-glial signaling (Eckhardt, 2008). ARSA deficient MLD patients consequently lack sufficient sphingolipids, thus leading to downstream myelin deficits (Biffi et al., 2006). Original MLD therapies included hematopoietic cell or bone marrow transplantations (BMT) but were limited in their therapeutic success (Krivit et al., 1999; Peters and Steward, 2003). Since then, use of lentiviruses and AAVs have proven more promising; LV treatment of microglial populations in the tremor mouse model has exhibited restorative functions (Biffi et al., 2006) and AAVrh10 carrying the ARSA transgene has been found to correct for MLD by targeting up to 21% of oligodendrocytes (Piguet et al., 2012). Likewise, Krabbe's disease gene therapy has promoted use of alternative glial targeting for restorative function and remyelination. KD is caused by the loss of function of the lysosomal enzyme galactoceramidase (GALC). Comparable to MLD treatments, primary KD therapy involved BMTs which provided limited success (Marshall et al., 2018). Use of AAV9 in two separate studies have demonstrated that ubiquitous CNS expression of GALC reduces disease-associated astrogliosis and microgliosis and furthermore extends the lifespan of KD mice (Marshall et al., 2018; Pan et al., 2019).

Notably, leukodystrophies such as CD, MLD, and KD result from enzyme deficiencies and therefore disease mitigation could involve forms of metabolic restoration *via* cross-correction approaches, either through enzyme redistribution by substitute cells or by metabolite recompartmentalization and/or processing by neighboring cells. Although metabolic cross-correction mechanisms have not yet been established in Canavan disease, it is possible that such strategies may be therapeutic for non-metabolic leukodystrophies. Therefore, the therapeutic achievements using gene therapy, as well as potentially metabolic treatments, to target alternative glial cell-types, further highlight the advantages of non-traditional cell type targeting for leukodystrophies on a restorative front.

## Conclusion

Leukodystrophies, which are largely classified according to the affected CNS cell-type in the respective white matter disorder (van der Knaap and Bugiani, 2017), remains elusive in concrete treatments for disease pathogenesis, in part due to variable disease onset, lack of prognosis screening, and neuropathological complexity associated with disease progression. These drawbacks can therefore lead to unsuccessful clinical intervention on a time-sensitive front, potentially resulting in significant, irreversible cognitive impairment and even fatality. For Canavan disease, in particular, a current absence of NAA's conclusive role in the CNS further compounds treatment applications for the leukodystrophy. In addition, complexities involved in selectively targeting oligodendrocyte populations limits the range of therapeutics for this disease which is thought to be predominately oligodendrocyte affected. Nonetheless, use of gene therapies, notably AAVs, have shown promising results in targeting other glial cell types, particularly astrocytes, as a restorative option for Canavan disease. Given the homeostatic role of astrocytes in the CNS, it is not completely unexpected that astrocyte-restricted ASPA expression leads to complete reversal of CD CNS pathology and sustained myelination and survival in CD mouse models. The implications of such research suggest that other leukodystrophies may benefit from gene therapy for alternative glial targeting, potentially non-oligodendrocyte focused, an area which is certainly worth delving into given the successes observed in Canavan disease research thus far.

## Author Contributions

AL outlined the review and wrote the manuscript. DG contributed to the outline of the review. DG and GG edited the manuscript. AL, DG, and GG prepared the paper. All authors contributed to the article and approved the submitted version.

## Conflict of Interest

GG is a co-founder of ASPA Therapeutics, Adrenas Therapeutics, and Voyager Therapeutics and holds equity in the companies. DG is a co-founder of ASPA Therapeutics and hold equity in the company. GG is an inventor of patents with royalties licensed to Voyager Therapeutics, ASPA Therapeutics, and other biopharmaceutical companies. DG is a member of the Clinical and Scientific Advisory Board of ASPA Therapeutics. GG and DG are inventors on a patent (PCT/US2016/058197) that is relevant to the content of this manuscript and may result in potential royalties if granted or licensed. The remaining author declares that the research was conducted in the absence of any commercial or financial relationships that could be construed as a potential conflict of interest.
